# Physicochemical Properties and Aroma Profiles of Golden Mulberry Fruits at Different Harvesting Stages

**DOI:** 10.3390/molecules30132717

**Published:** 2025-06-24

**Authors:** Kunfeng Li, Wen Tan, Lingxia Huang, Jinhu Tian

**Affiliations:** 1 Agricultural Experimental Station, Zhejiang University, Hangzhou 310058, China; 0016554@zju.edu.cn; 2 College of Biosystem Engineering & Food Science, Zhejiang University, Hangzhou 310058, China; wentan0306@163.com; 3 Key Lab Silkworm & Bee Resource Utilization & Innovation, Zhejiang Univerisity, Hangzhou 310058, China; 4 Zhejiang University Zhongyuan Institute, Zhengzhou 450001, China; jinhutian@126.com; 5 Wuxi Xishan Modern Agriculture Joint Research Center, Zhejiang University, Wuxi 214117, China

**Keywords:** golden mulberry, harvesting time, fruit quality, aroma component

## Abstract

Golden mulberry (*Morus macroura* Miq.) is favored for its rich bioactive components and unique flavor, but fruit quality depends on harvest time. In the present study, golden mulberry fruits were collected at 18 (T1), 21 (T2), 24 (T3), and 27 (T4) days after flowering to investigate the impact of the harvesting stage on its physicochemical properties, antioxidant capacity, and aroma profile. Physicochemical parameters such as total phenols, total soluble solids, titratable acidity, and sensory evaluation revealed that the hardness gradually decreased with fruit maturity, whereas the weight of single fruit, total soluble solids, and solid–acid ratio increased, and soluble sugars, titratable acidity, total polyphenols and sugar–acid ratio initially increased and then decreased. Antioxidant capacity, measured by ABTS, FRAP, and DPPH assays, decreased with ripening, but stabilized at T3. In addition, the aroma components of golden mulberry fruit were analyzed by GC-MS, and it was found that aldehyde, alcohol, and ester were the main aroma components of the golden mulberry fruit. Combining the physicochemical indices, sensory evaluation, and aroma profiles, T3 period considered the optimal harvesting time. These findings offer practical guidance for the optimal harvesting and utilization of golden mulberry fruits.

## 1. Introduction

*Fructus mori*, the pharmacopeial term for mulberry fruits, is rich in bioactive compounds, including amino acids, anthocyanins, alkaloids, polysaccharides, polyphenols, and other nutrients [1,2]. These components confer a range of pharmacological benefits, including antioxidant, anti-aging, immune-enhancing, and anticancer effects, etc. [3,4,5]. Additionally, mulberry fruits have been shown to lower blood glucose, blood pressure, and blood lipid levels, earning them the reputation as a “superior health fruit” of the 21st century [6]. In recent years, with the continuous enrichment of mulberry varieties and the innovation and development of cultivation modes, mulberry fruit agriculture, which mainly focuses on fresh fruit, has been rapidly growing [7,8,9]. As an emerging economic fruit, the current research on mulberry fruit mainly focuses on its nutritional composition [10], medicinal value [11], and cultivation management [12,13]. However, few studies have been conducted on the effect of mulberry fruit maturity on its quality (e.g., texture, antioxidant activity, flavor, etc.).

Fruit quality primarily includes external attributes such as shape, color, and weight, as well as internal characteristics like sugar content, acidity, vitamins, and aroma compounds [14]. Significant differences in appearance quality, nutrient composition, and aroma profile have been observed at different harvesting stages, directly influencing consumer preferences [15,16]. Moreover, the harvesting stage is a critical determinant of fruit storability [17,18]. Studies have shown that harvesting at early maturity can result in poor coloration and insufficient accumulation of nutrients and aroma compounds [19]. Conversely, harvesting at an overly mature stage increases the risk of browning, decay, and other forms of quality deterioration, along with reduced storage resistance, ultimately shortening the shelf life [20,21]. Therefore, selecting the appropriate maturity stage is essential to ensure optimal fruit quality and postharvest performance.

Golden mulberry (*Morus macroura* Miq.), also known as Himalayan white long-fruited mulberry, is a high-quality cultivar originally native to the Pakistan region. In recent years, it has gained popularity for fresh consumption. Compared with traditional black and red mulberry cultivars, golden mulberry fruits exhibit a softer coloration, an elongated shape, and a sweet, yet non-cloying taste. Notably, when fully ripe, they generate a distinctive creamy flavor and have become one of the most popular varieties for fresh eating and recreational picking. At present, studies on golden mulberry in China are limited and have mainly focused on cultivation and orchard management techniques [22]. However, there is a lack of research on the relationship between fruit maturity and quality across different developmental stages. This has resulted in aimless harvesting practices, making it challenging to ensure consistent fruit quality. Therefore, the present study used golden mulberry as the experimental material to systematically analyze the differences in appearance quality, physicochemical properties, and aroma compounds at different harvesting stages, with the aim of establishing scientific criteria for fruit quality evaluation and providing a basis for determining the optimal harvest time for golden mulberry.

## 2. Results

### 2.1. Impact of Harvesting Time on Fruit Drop, Mold Incidence, and Quality of Golden Mulberry Fruit

To investigate the effects of different harvesting time on mulberry fruit quality, samples were classified into four developmental stages based on the number of days after flowering—T1 (18 days), T2 (21 days), T3 (24 days), and T4 (27 days)—representing progressively ripening stages used for subsequent analyses. As shown in Table 1, with delays in harvesting, the fruit drop percentage and mold percentage of mulberry fruits showed an overall increasing trend and the marketable fruit percentage showed a decreasing trend, with the latter declining the most seriously at the T4 yellow–white period (27 days after flowering), which was only 65.7% ± 3.21%, in which the percentage of fruit drop was the main reason affecting the quality of mulberry fruits in the T4 period.

### 2.2. Effect of Harvesting Time on Fruit Shape Index and Single Fruit Weight of Golden Mulberry

As shown in Table 2, the single-fruit weight of golden mulberry significantly increased from T1 to T3 (*p* < 0.05), with no significant difference observed between T3 and T4 (*p* > 0.05). The fruit shape index decreased significantly from T1 to T3 (*p* < 0.05), but remained relatively stable between T3 and T4 (*p* > 0.05). These results suggest that the fruit weight and shape index plateaued between T3 and T4, indicating stabilization of fruit quality parameters during these stages.

### 2.3. Effect of Harvesting Time on the Hardness and Color Characteristics of Golden Mulberry Fruit

Hardness is a key indicator of fruit maturity, affecting fruit quality, storage, and sensory attributes. As shown in Table 2, the hardness of golden mulberry fruit decreased with maturity and there were significant differences among the different harvesting times (*p* < 0.05), indicating that the texture of the fruit gradually softened with maturity. Color is also a key indicator of the maturity of mulberry fruit. Many common mulberry varieties, such as black and red mulberries, typically develop a purple or purple–black hue with age. However, as a newly introduced variety, the color of golden mulberry fruits differs significantly from that of common purple mulberries. The immature fruit starts from green and gradually transitions to yellow–green and yellow–white as it ripens. As shown in Table 2, as the maturity increased, the value of L* increased continuously, indicating that the color of the fruit gradually became lighter. The value of a* decreased and then increased from T1 to T4, and the value of b* and its chromaticity increased and then decreased from T1 to T4, reflecting a color shift from greenish to bright yellow and ultimately to yellowish white. C* represents color saturation, which tends to increase and then decrease during the ripening process, indicating that the fruit has the most vivid color during the mid-ripening stage (T3) and a slightly softer color after full ripening (T4).

### 2.4. Sensory Evaluation of Golden Mulberry Fruit at Different Harvesting Stages

As shown in Table 3, the color and flavor of the golden mulberry fruit significantly increased from T1 to T3 (*p* < 0.05), with the difference becoming smaller from T3 to T4. Juiciness and sweetness showed a significant increasing trend from T1 to T4 (*p* < 0.05), while sourness exhibited a significant decreasing trend (*p* < 0.05). In terms of the overall sensory score, T3 had the highest score of 79.68 ± 1.27, followed by T4 and T2 with scores of 73.17 ± 1.91 and 67.98 ± 1.31, respectively. The lowest score was observed in T1, with a score of only 46.84 ± 1.99. Therefore, the sensory evaluation for T3 was the highest, indicating the best flavor, while T4 and T2 had slightly lower flavor quality. T1, with the lowest score, was unsuitable for fresh harvesting.

### 2.5. Impact of Harvesting Time on the Nutritional Quality of Golden Mulberry Fruit

Total soluble solids (TSSs), soluble sugars (SSs), and titratable acidity (TA) are key physicochemical indicators for assessing fruit ripeness, nutritional quality, and organoleptic characteristics. These parameters reflect the dynamic accumulation of sugars, organic acids, vitamins, amino acids, and minerals that occur during the fruit maturation process [23]. As shown in Table 4, with the increasing maturity of golden mulberry fruit, the TSS content gradually increased, with significant differences among harvesting times (*p* < 0.05). The TSS content reached its highest value of 26.19% during the T4 period. The SS content exhibited an initial increase followed by a decline, peaking at T3 and then decreasing at T4. The SS content at T3 was significantly higher than that at other stages (*p* < 0.05). Titratable acidity (TA), on the other hand, showed a decreasing trend from T1 to T3, followed by a slight increase at T4, with the highest value at T1 and the lowest at T3. The total phenolic content first decreased and then increased, with the greatest decrease observed between T2 and T3, where it decreased by 73.56%. From T3 to T4, the total phenolic content slightly increased, but remained significantly lower than that of T1 and T2 (*p* < 0.05). The TSS/TA ratio increased significantly from T1 to T3 (*p* < 0.05), with no significant difference between T3 and T4. The SS/TA ratio increased significantly from T1 to T3 (*p* < 0.05) and then decreased significantly from T3 to T4 (*p* < 0.05), with the highest value observed at T3. Based on the combined sensory evaluation, appearance quality, and internal nutritional quality analysis, the T3 period was considered the most suitable for fresh harvesting, as it showed the most stable fruit shape index, highest sensory score, SSs, and SS/TA ratio.

### 2.6. Antioxidant Capacity of Golden Mulberry Fruit at Various Harvesting Stages

**Figure 1:**
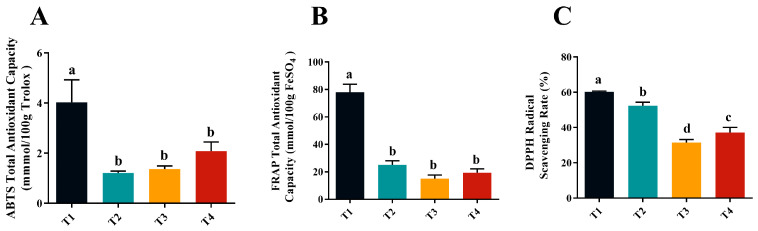
Effects of maturity on antioxidant capacity of golden mulberry. (**A**) ABTS, (**B**) FRAP, and (**C**) DPPH. Different letters indicate that significantly difference at 0.05 level.

As shown in Figure 1A,B, the antioxidant activity of golden mulberry fruit determined by both 2,2′-azino-bis (3-ethylbenzothiazoline-6-sulfonic acid) (ABTS) and ferric reducing antioxidant power (FRAP) methods showed that the highest total antioxidant capacity was found in the T1 phase, with a value of 4.03 mmol TE/100 g dry weight (DW) and 78.07 mmol Fe^2+^ equivalents per 100 g DW, respectively. These values were significantly higher than those of the T2, T3, and T4 phases (*p* < 0.05), with no significant differences among the T2, T3 and T4 phases (*p* > 0.05). Figure 1C shows that the free radical scavenging activities of golden mulberry fruits were in the order of T1 > T2 > T4 > T3, with significant differences between different harvesting times. This pattern is consistent with the findings of previous studies, where researchers also reported that phenolic compounds were the major antioxidants in mulberry, which was further confirmed by the trends in 1-diphenyl-2-picrylhydrazyl radical (DPPH) scavenging and total phenolic content [24,25].

### 2.7. Analysis of Aroma Components in Golden Mulberry Fruit Across Different Harvesting Stages

A total of 67 volatile flavor substances were annotated by GC-MS analysis in four different periods of golden mulberry fruits, among which aldehydes were the most abundant (16), followed by alcohols (15), esters (12), alkanes (5), ketones (4), furans (4), acids (3), alkenes (3), dimethyl hydroxylamine, 1,2,4-triazole, 1-methyl-1,2,4-triazole, 4-methoxybiphenyl, allyl butyl ether, and other substances. As shown in Table 5, there were significant differences in the types and composition of volatile substances annotated in the mulberry fruits harvested from different periods. A total of 52 volatile compounds were annotated in T1, 46 in T2, 47 in T3, and 41 in T4, among which methyl nonanoate, allyl oxalate, methyl palmitate, methyl caproate, cis-2-penten-1-ol, 1-penten-3-ol, 2-methoxy-pentane, 1-penten-3-one, n-hexanal, nonanal, 2-hexenal, 2-pentylfuran, 2-butyltetrahydrofuran, and other volatiles were annotated at all four maturity levels. Substances specific to the T1 period included tetrahydrofurfuryl acrylate, 2-methyl-3-buten-1-ol, n-pentanol, 1-azabicyclo [3.1.0] hexane, trans-2-pentenal, 2-methyl-2-buten-2-ol, n-pentenyl alcohol, and n-pentanol. Pentenal, 2,2-dimethyl-4-octenal, and propionic anhydride were the most abundant endemic substances, but their content accounted for only 2.4% of the total content in the T1 period of harvesting. 4,5-dimethyl-2-hepten-3-ol, 6,6-dimethyl-1,3-heptadien-5-ol, 3-methoxy-1-hexene, and dimethyl hydroxylamine were endemic to the T2 period and accounted for 6.7% of the T2 period’s total content. The content of 2-methylcyclopentanol and 2-methylhept-6-en-3-one in the T3 period accounted for only 1.9% of the T3 period, and no specific substances were found in the T4 period, indicating that the relative content of the specific flavor substances annotated in the T4 period was relatively little and might show little influence on the flavor of the mulberry fruits.

**Figure 2:**
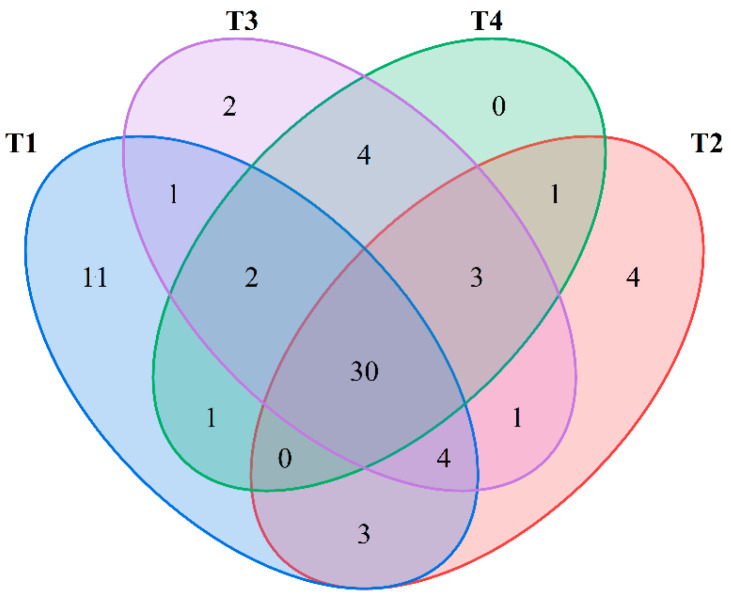
Common and unique aroma components in golden mulberry.

To further demonstrate the differences in the aroma components of mulberry fruits across the four maturity periods, a Venn diagram was applied to depict the number of shared and unique characteristic aromas at different maturity stages. As shown in Figure 2, 67 aroma components were annotated in mulberry fruits during the four periods, of which 30 were common components. Only the T4 period had no unique components, whereas the other three periods had unique components. The T1 period had 11 kinds of unique components, with T2 and T3 having 4 and 2 unique components, respectively.

**Figure 3:**
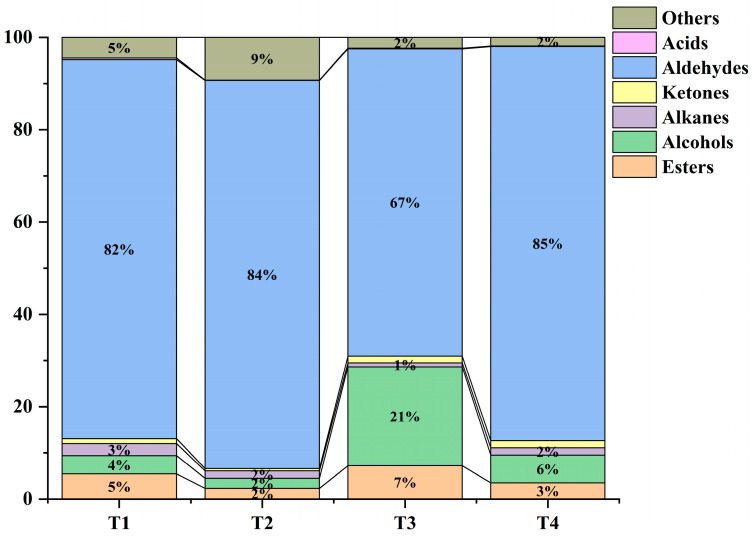
Relative content of aroma components of golden mulberry at different maturity stages.

As shown in Figure 3, the aroma components of mulberry fruit in different periods were mainly acids, aldehydes, ketones, alkanes, alcohols, and esters. Among them, aldehydes were the dominant aroma components, followed by alcohols and esters. The sum of the relative content of aldehydes, alcohols, and esters in the four periods was 91%, 88%, 95%, and 94%, respectively. The relative aldehyde content was the lowest in the T3 period, but the relative alcohol and ester content was the highest in this period. The relative aldehyde content was close to that in the T2 and T4 periods, and the relative alcohol and ester content was lower than that in the T4 period. The aldehyde content at T2 and T4 was similar, and the relative alcohol and ester content was lower at T4.

## 3. Discussion

In the present study, different harvesting times of golden mulberry fruit were used to investigate their effects on the marketable percentage, appearance quality, and physical and chemical qualities of the golden mulberry fruit. With the increase in maturity, the percentage of fruit drop and mold percentage was significantly increased, resulting in a significant decline in the percentage of marketable fruit. The T3 period still had 89.0% marketable fruit percentage, but the percentage of marketable fruit declined more seriously in the T4 period, accounting for only 65.7%. Therefore, from an economic perspective, it is advisable to harvest mulberry fruits before the T3 stage.

The quality indices of TSSs, SSs, and total acid are important indicators for evaluating the postharvest quality and flavor of mulberry fruits [26,27]. In the present study, further analysis of the quality indices of TSSs, total sugar, and total acid of golden mulberry fruits in different periods showed that the indices of total sugar, total acid, and SSs/TA all reached a balance in the period of T3, with total sugar and SSs/TA having the highest values and total acid the lowest value. Although the value of TSSs had reached a maximum of 26.19 ± 0.91 at T4, the corresponding mulberry fruits at this time had a mushy texture and might not be suitable for eating. The content of TSSs, total acid, and solid–acid ratio are commonly used to evaluate the flavor of fruits [28], but none of those indicators considered the degree of contribution of specific sugar–acid fractions, and therefore did not reflect the true sweet and sour flavor of fruits, especially when the sugar content of fruits was at a high level, and fruits with appropriate acidity tended to be more accepted by consumers [29]. The sensory evaluation results (Table 3) showed that the T3 fruit received the highest scores in flavor, color, and overall sensory quality, indicating a better perceived balance between sweetness and acidity. Therefore, by comprehensively analyzing the physicochemical parameters (TSSs, total sugar, total acid, SSs/TA) and sensory evaluation results, it can be concluded that the golden mulberry fruit harvested at the T3 stage exhibited the most balanced sweet and sour flavor and optimal overall eating quality.

The process of free radical scavenging in plants is complex and closely related to antioxidant activity [30,31]. To comprehensively assess the antioxidant activity of mulberry fruits at different ripening stages, three methods, ABTS, FRAP, and DPPH, were used in the present study. The results showed that all mulberry fruits at all harvesting stages possessed antioxidant activity, with the strongest free radical scavenging capacity at T1 and non-significant differences at T2, T3, and T4. The trends of total polyphenol content and DPPH values in the present study were consistent with the study of Makavelou et al. [32], who reported that total anthocyanin and polyphenol content in mulberry genotypes was significantly associated with higher antioxidant capacities (FRAP and DPPH) and that anthocyanins were the primary contributors to the total antioxidant activity. Suriyaprom et al. analyzed the antioxidant capacity of mulberry fruit extract and found that the total phenol, flavonoid, and anthocyanin content in mulberry fruit was significantly correlated with its antioxidant capacity [31]. Polyphenols exert antioxidant activity through the structural characteristics of their phenolic hydroxyl groups. These hydroxyl groups can effectively scavenge free radicals by donating hydrogen atoms or electrons. By comparing the changes in the antioxidant capacity of mulberries at different maturity stages, this study further validated the relationship between mulberry maturity and antioxidant activity. As the maturity of mulberries increase, changes in total polyphenol content may be one of the main factors contributing to the differences in antioxidant capacity.

The most important characteristic of golden mulberry is its milk flavor, which is also an important index for determining the quality of golden mulberry fruit. In the present study, GC-MS analysis revealed significant differences in the types of components annotated in the mulberry fruits at different harvesting times and the content of the same components at different periods. Aldehyde, alcohol, and ester are the dominant substances in golden mulberry at different periods [33]. In addition, aldehydes were highest in terms of species and relative content, followed by alcohols and esters. The combined relative content of these three groups of compounds exceeded 90% in all periods except T2, where it slightly fell to 88%. In the other three periods, the sum of the aldehyde, alcohol, and ester content remained above 90%. These results were consistent with the study of Jia et al., who found that the major components of nine mulberry varieties were aldehydes, esters, and alcohols in that order [34]. The analyses also revealed that T3 had the lowest relative aldehyde content and the highest alcohol and ester content, suggesting that T3 was the critical stage of mulberry fruit ripening, while T4 was overripe. This finding aligned with the results of the sensory evaluation, SS, and SS/TA analyses in this study.

## 4. Materials and Methods

### 4.1. Materials

Sodium hydroxide, DPPH, and glucose standard were purchased from Shanghai Maclin Biochemical Technology Co., Ltd. (Fengxian, Shanghai, China). 2-Octanol standard (CAS No. 6169-06-8) was obtained from Shanghai Aladdin Biochemical Technology Co., Ltd. (Pudong, Shanghai, China). Acetonitrile (HPLC grade) and formic acid (HPLC grade) were purchased from Sigma-Aldrich Corporation (Milwaukee, WI, USA). The total antioxidant capacity assay kits (ABTS method and FRAP method) were purchased from Shanghai Biyuntian Biotechnology Co., Ltd. (Shanghai, China).

### 4.2. Sample Preparation

**Figure 4:**
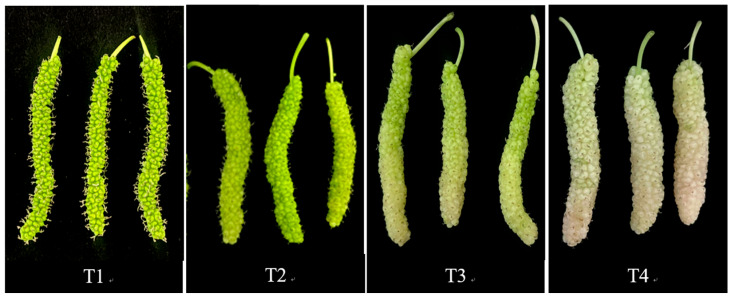
Photographs of golden mulberry fruits at different harvesting times. T1, 18 d after flowering. T2, 21 d after flowering. T3, 24 d after flowering. T4, 27 d after flowering.

The test varieties of golden mulberry (*Morus macroura* Miq.) were harvested in 2024 from the No. 3 facility greenhouse in the Agricultural Science and Technology Park of Zhejiang University, Changxing County, Huzhou City, Zhejiang Province. The plants were cultivated under controlled conditions with daytime temperature 25 ± 3 °C, night temperature 18 ± 3 °C, relative humidity 60%–75%, and soil pH 6.5. The fruit development period and peel color were evaluated at T1, T2, T3, and T4 after flowering. At each time point, 1000 g of samples was collected, and 30 mulberries were randomly selected to measure the longitudinal diameter, transverse diameter, and individual fruit mass (Figure 4). The remaining samples were quickly frozen in liquid nitrogen and stored at −80 °C for the determination of other indices.

### 4.3. The Percentage of Mulberry Fruit Drop, Mold, and Marketable Fruit

The fruit drop percentage, mold percentage, and marketable fruit percentage are essential indicators of the mulberry’s quality [16]. The percentage of fruit drop was calculated based on the total number of mulberries and the number of fruits that had detached and fallen from the tree. The percentage of mold was determined by counting the number of moldy or spoiled fruits remaining on the tree. The marketable fruit percentage was calculated as the percentage of the total number of fruits minus the dropped fruits and moldy or spoiled fruits.

Fruit Drop percentage = Number of Fallen Fruits/Total Number of Fruits × 100(1)

Mold percentage = (Number of Moldy Fruits/Total Number of Fruits) × 100 (2)

Marketable Fruit percentage = 100% − Fruit Drop percentage − Mold percentage(3)

### 4.4. Measurement of Appearance Indicators

A Vernier caliper was used to measure the longitudinal diameter (mm) and transverse diameter (mm) of the fruits, and the ratio of the longitudinal diameter of the fruits to the transverse diameter of the fruits was used to express the fruit shape index.

A total of 30 mulberries were randomly selected at each stage to determine their mass at various developmental stages. Measurements were conducted using an electronic balance, with 10 fruits weighed per batch. The average mass of a single fruit was calculated and expressed in grams (g).

Fruit hardness was assessed using a fruit hardness tester equipped with a probe diameter of 3.53 mm and a penetration depth of 1.00 mm. Measurements were performed on three randomly selected mulberries of uniform maturity, targeting the equatorial region of the fruits. The results are expressed in kg/cm^2^.

The equatorial region of the fruit was measured with a colorimeter (UltraScan VIS, HunterLab, Reston, VA, USA.) with a 1 cm aperture [35]. The color parameters L* (brightness index), a* (red saturation index), and b* (yellow saturation index) were recorded [36]. Chromaticity (C*) was calculated using the following formula:

(4) $$\mathrm{Chromaticity}(C*)=\sqrt{{a*}^{2}+{b*}^{2}}$$

The sensory evaluation was conducted using the method reported by Feng et al., with appropriate modifications based on the specific circumstances of this study [37]. Twelve experienced sensory evaluators (five males and seven females) aged between 20 and 28 years, all with prior experience in sensory evaluation of fruit and vegetable products, were recruited. Prior to the formal evaluation, they received standardized training on sample characteristics, scoring criteria, and operational procedures to ensure the accuracy and consistency of the evaluation results. The specific scoring criteria are detailed in Table 6.

### 4.5. Material Composition Analysis

Ten uniformly sized and randomly selected fruits were crushed into a homogenate and then filtered through a 100-mesh cloth to obtain 1 mL of the filtrate, and the TSS content was measured and recorded using a portable refractometer (Atago, Tokyo, Japan). SSs were measured using the anthrone reagent method, in which the sample was reacted with anthrone reagent under acidic conditions, producing a green–blue complex that was measured spectrophotometrically at 620 nm [37]. TAs were determined by titrating the sample with a standardized NaOH solution until reaching a neutral pH endpoint according to the method described by Morris et al. [38]. Total phenolic content was assessed using the Folin–Ciocâlteu colorimetric assay, where phenolic compounds reduced the Folin–Ciocâlteu reagent to form a blue complex, quantified by absorbance at 765 nm, and expressed as gallic acid equivalents (GAEs) [4].

### 4.6. Evaluation of Antioxidant Activity

Briefly, 0.1 g freeze-dried mulberry fruit powder with different maturity levels was added to 2 mL of anhydrous ethanol, incubated in a water bath at 50 °C for 2 h, and then centrifuged at 4000 rpm for 15 min using an Eppendorf 5424 R centrifuge (Hamburg, Germany) equipped with an FA-45-24-11 rotor, and the supernatant was extracted for measurement. The DPPH radical scavenging activity of the extract was measured at 510 nm using a microplate [39], where the antioxidant compounds reduced the stable DPPH radical, causing a decrease in absorbance proportional to radical scavenging capacity. Total antioxidant capacity was evaluated by the ABTS assay at 734 nm using Trolox as a standard [40], which involves generation of the ABTS^+^ radical cation and measurement of its reduction by antioxidants in the sample. The FRAP of the extract was determined at 593 nm with FeSO_4_ as a reference [41] based on the reduction of Fe^3+^ to Fe^2+^ by antioxidants, forming a colored ferrous-tripyridyltriazine complex that was quantified spectrophotometrically.

### 4.7. Determination of Aroma Composition

Mulberry fruit stored at −80 °C was ground to a powder using liquid nitrogen. An accurate weight of 2 g was placed in a 20 mL headspace bottle containing 1 mL of saturated sodium chloride solution and 1 g sodium chloride solids. Then, 10 μL of 2-octanol (concentration: 100 μg/mL) was added. The cap was quickly tightened, and the mixture was subjected to ultrasonic treatment for 30 min. Finally, the solution was filtered through a 0.22 μm membrane to obtain the sample for testing.

The analysis was conducted using an Agilent 7250 GC/Q-TOF instrument (Santa Clara, CA, USA) equipped with a DB-5MS column (30 m × 250 μm × 0.25 μm). The extraction conditions included a pre-aging time of 5 min at 250 °C, followed by an incubation time of 15 min at 50 °C. The sample extraction was performed for 30 min, with a desorption time of 5 min. An Arrow SPME DVB/PDMS 20 mm, 120 μm extraction head (Agilent, Santa Clara, CA, USA) was used. Chromatographic conditions involved a constant flow rate of 1.0 mL/min with a 25:1 shunt ratio. The heating program began at 40 °C, was held for 3 min, and then increased at a rate of 10 °C/min to 250 °C. For mass spectrometry, the scanning range was set from *m*/*z* 30 to 450, and the ion source temperature was maintained at 230 °C.

C_i_ = (A_i_ × C_0_ × V_0_)/(A_0_ × M) (5)

where, C_i_ is the mass concentration of the compound to be tested (μg/g). A_i_ is the peak area of the compound to be tested. C_0_ is the mass concentration of the compound added (μg/mL). V_0_ is the volume of the compound added to the internal standard (mL). A_0_ is the peak area of the compound added to the internal standard, and M is the mass of the sample injected each time (g) [42].

### 4.8. Statistical Analysis

All measurements were performed in triplicate and are expressed as means ± standard deviation (SD). Data were analyzed using Excel 2019. Statistical significance was determined using one-way analysis of variance (ANOVA) and independent-sample *t*-tests using SPSS software (version 27.0, IBM Corp., Armonk, NY, USA). Differences were considered statistically significant at *p* < 0.05. Graphing was performed using Origin 2024 and GraphPad prism 9.5 software.

## 5. Conclusions

In conclusion, the T3 stage of golden mulberry exhibited the best overall quality, highest fruit yield, a well-balanced sweet and sour flavor, and favorable sensory attributes. This stage also had the most optimal flavor composition, with aroma compounds (aldehydes, alcohols, and esters) accounting for over 95% of the volatile components. The highest total antioxidant capacity was recorded in the T1 stage (4.03 mmol TE/100 g DW and 78.07 mmol Fe^2+^ equivalents per 100 g DW), significantly higher than that of the T2, T3, and T4 stages (*p* < 0.05), with no significant differences among T2, T3, or T4. Although TSS content was highest in the T4 stage (26.19 ± 0.91), the fruit was overly ripe, which negatively affected its texture and taste, making it mushy and unsuitable for consumption. In contrast, the T3 stage yielded an optimal balance of total sugars, SS/TA ratio, and aroma, making it the ideal stage for harvesting golden mulberries for fresh consumption. These findings provide a scientific basis for harvesting mulberries at various stages of ripeness based on their intended use.

## Figures and Tables

**Table 1:** The percentage of fruit drop, mold and marketable fruit of golden mulberry at different harvesting times.

Group	Fruit Drop (%)	Mold (%)	Marketable Fruit (%)
T1	1.30 ± 0.60 ^d^	0.00 ± 0.00 ^c^	98.73 ± 0.64 ^a^
T2	3.30 ± 0.60 ^c^	0.00 ± 0.00 ^c^	96.75 ± 0.61 ^b^
T3	9.30 ± 1.50 ^b^	1.72 ± 0.16 ^b^	89.01 ± 1.72 ^c^
T4	28.71 ± 2.11 ^a^	5.72 ± 1.20 ^a^	65.73 ± 3.21 ^d^

Note: Data in the table are expressed as means ± standard deviation. For each line, different lowercase letters correspond to values significantly different at *p* < 0.05.

**Table 2:** Effect of maturity on size, shape, firmness, and color of golden mulberry.

Indicator	Time			
	T1	T2	T3	T4
Single-fruit weight/g	11.97 ± 0.80 ^c^	18.07 ± 0.95 ^b^	21.39 ± 1.91 ^a^	22.18 ± 1.45 ^a^
Fruit shape index	10.25 ± 0.92 ^a^	8.01 ± 0.79 ^b^	6.75 ± 0.73 ^c^	6.67 ± 0.63 ^c^
Hardness/N	2.18 ± 0.04 ^a^	1.48 ± 0.28 ^b^	0.66 ± 0.07 ^c^	0.32 ± 0.04 ^d^
L*	25.24 ± 1.03 ^c^	39.54 ± 2.48 ^c^	44.82 ± 0.33 ^a^	44.59 ± 1.16 ^a^
a*	−7.52 ± 0.17 ^b^	−9.14 ± 0.10 ^c^	−9.61 ± 0.18 ^c^	−4.77 ± 0.22 ^a^
b*	11.95 ± 0.40 ^d^	18.10 ± 0.89 ^b^	19.90 ± 0.41 ^a^	15.07 ± 0.02 ^c^
C*	14.08 ± 0.44 ^d^	19.98 ± 0.64 ^b^	22.04 ± 0.46 ^a^	15.80 ± 0.09 ^c^

Note: Data in the table are expressed as means ± standard deviation. For each line, different lowercase letters correspond to values significantly different at *p* < 0.05.

**Table 3:** Effect of maturity on the sensory score of golden mulberries.

Indicator	Time			
	T1	T2	T3	T4
Color	14.15 ± 0.26 ^d^	17.38 ± 0.51 ^c^	18.68 ± 0.77 ^a^	16.92 ± 0.64 ^b^
Juiciness	2.90 ± 0.31 ^d^	13.62 ± 0.28 ^c^	17.45 ± 0.75 ^b^	19.35 ± 0.23 ^a^
Sweetness	8.43 ± 0.21 ^d^	12.43 ± 0.47 ^c^	18.67 ± 0.25 ^b^	19.27 ± 0.16 ^a^
Sourness	18.38 ± 0.29 ^a^	14.90 ± 1.27 ^b^	6.60 ± 0.43 ^c^	2.27 ± 0.34 ^d^
Flavor	2.98 ± 0.23 ^d^	9.65 ± 0.23 ^b^	18.28 ± 0.28 ^a^	14.93 ± 0.63 ^b^
Total score	46.84 ± 1.99 ^d^	67.98 ± 1.31 ^c^	79.68 ± 1.27 ^a^	73.17 ± 1.91 ^b^

Note: Data in the table are expressed as means ± standard deviation. For each line, different lowercase letters correspond to values significantly different at *p* < 0.05.

**Table 4:** Effect of maturity on compositional attributes of mulberry.

Indicator	Time			
	T1	T2	T3	T4
TSSs (%)	6.04 ± 0.54 ^d^	14.8 ± 0.67 ^c^	17.11 ± 0.57 ^b^	26.19 ± 0.91 ^a^
SSs (mg/g)	113.21 ± 3.15 ^d^	296.93 ± 5.20 ^b^	349.79 ± 9.04 ^a^	282.06 ± 8.04 ^c^
TA (mg/g)	26.62 ± 0.77 ^a^	19.71 ± 2.95 ^b^	13.61 ± 1.39 ^c^	19.93 ± 1.66 ^b^
Total phenolic content (mg GAE/g)	3.75 ± 0.02 ^a^	3.67 ± 0.02 ^b^	0.97 ± 0.03 ^d^	1.24 ± 0.06 ^c^
TSSs/TA	0.25 ± 0.03 ^c^	0.75 ± 0.01 ^b^	1.29 ± 0.06 ^a^	1.32 ± 0.04 ^a^
SSs/TA	4.22 ± 0.06 ^d^	20.06 ± 0.45 ^b^	25.70 ± 1.57 ^a^	14.22 ± 0.19 ^c^

Note: Data in the table are expressed as means ± standard deviation. For each line, different lowercase letters correspond to values significantly different at *p* < 0.05.

**Table 5:** Composition and content of volatile substances in golden mulberry of different maturity.

Number	Category	Compound Name	CAS	Content/(μg/kg)			
				T1	T2	T3	T4
1	**esters**	Octanoic acid, methyl ester	111-11-5	77.07 ± 16.70 ^a^	17.25 ± 0.06 ^b^	4.69 ± 2.70 ^b^	6.15 ± 0.23 ^b^
2		Butyrolactone	96-48-0	60.48 ± 0.00	-	-	-
3		Tetrahydrofurfuryl acrylate	2399-48-6	53.72 ± 0.00	-	-	-
4		2-Propenoic acid, 2-methyl-, octyl ester	2157-01-9	38.20 ± 1.13 ^a^	-	7.40 ± 0.00 ^b^	5.57 ± 2.54 ^b^
5		Nonanoic acid, methyl ester	1731-84-6	36.85 ± 2.30 ^a^	7.61 ± 0.32 ^b^	5.32 ± 0.95 ^b^	6.30 ± 0.48 ^b^
6		Oxalic acid, diallyl ester	615-99-6	26.68 ± 0.00 ^b^	5.09 ± 0.00 ^b^	131.68 ± 1.31 ^a^	-
7		n-Caproic acid vinyl ester	3050-69-9	20.75 ± 2.77 ^a^	-	2.89 ± 0.00 ^b^	2.88 ± 0.00 ^b^
8		Hexadecanoic acid, methyl ester	112-39-0	19.60 ± 0.15 ^a^	3.82 ± 0.50 ^b^	1.29 ± 0.00 ^c^	2.14 ± 0.73 ^c^
9		Ethyl (E)-hex-3-enyl carbonate	175397-71-4	186.79 ± 10.24 ^a^	35.11 ± 8.28 ^b^	14.82 ± 7.31 ^b^	10.17 ± 2.03 ^b^
10		Heptanoic acid, methyl ester	106-73-0	17.82 ± 0.56 ^a^	4.01 ± 0.81 ^b^	1.72 ± 0.88 ^c^	4.11 ± 0.44 ^b^
11		Hexanoic acid, methyl ester	106-70-7	145.22 ± 18.48 ^a^	47.99 ± 3.85 ^b^	18.77 ± 12.90 ^b^	26.41 ± 0.63 ^b^
12		3-Hexen-1-ol, propanoate, (Z)-	33467-74-2	-	25.24 ± 3.62 ^a^	0.53 ± 0.00 ^b^	6.52 ± 0.00 ^b^
13	**alcohols**	4-Heptenal, (Z)-	6728-31-0	37.72 ± 3.81 ^a^	22.05 ± 0.00 ^b^	-	-
14		3-Buten-1-ol, 2-methyl-	4516-90-9	35.49 ± 0.00	-	-	-
15		Cyclohexanol, 1-ethenyl-	1940-19-8	25.42 ± 0.00 ^a^	3.48 ± 0.00 ^b^	-	-
16		Cyclobutene-3,4-diol, tetramethyl-	90112-64-4	15.46 ± 0.00	-	-	-
17		3-Cyclohexen-1-ol, 4-methyl-1-(1-methylethyl)-, (R)-	20126-76-5	14.00 ± 1.35 ^a^	3.40 ± 0.00 ^b^	1.20 ± 0.00 ^c^	1.02 ± 0.15 ^c^
18		2-Undecen-4-ol	22381-86-8	13.80 ± 4.27 ^a^	4.89 ± 1.45 ^b^	2.07 ± 1.14 ^b^	1.43 ± 0.00 ^b^
19		2-Penten-1-ol, (Z)-	1576-95-0	121.58 ± 6.98 ^a^	26.48 ± 0.64 ^b^	7.85 ± 5.85 ^c^	7.35 ± 0.53 ^c^
20		1-Penten-3-ol	616-25-1	117.32 ± 2.58 ^a^	29.25 ± 1.08 ^b^	8.64 ± 7.09 ^c^	8.89 ± 0.83 ^c^
21		1-Octen-3-ol	3391-86-4	13.62 ± 1.33 ^b^	8.87 ± 0.93 ^b^	6.99 ± 5.62 ^b^	36.38 ± 2.47 ^a^
22		2-Heptanol, (S)-	6033-23-4	102.72 ± 0.00	-	-	-
23		2-Hepten-3-ol, 4,5-dimethyl-	55956-37-1	-	26.08 ± 0.00	-	-
24		6,6-Dimethyl-1,3-heptadien-5-ol	81912-03-0	-	15.87 ± 0.00	-	-
25		1-Hexanol	111-27-3	-	-	48.70 ± 6.22 ^b^	54.02 ± 1.79 ^a^
26		Cyclopentanol, 2-methyl-	24070-77-7	-	-	479.06 ± 0.00	-
27		Cyclohexanol	108-93-0	-	-	8.28 ± 1.36 ^b^	12.53 ± 0.00 ^a^
28	**alkanes**	Cyclopropane, propyl-	2415-72-7	164.29 ± 6.67 ^a^	94.09 ± 2.14 ^b^	-	-
29		1-Azabicyclo[3.1.0]hexane	285-76-7	149.44 ± 7.07	-	-	-
30		Pentane, 2-methoxy-	6795-88-6	14.03 ± 0.00 ^a^	4.93 ± 0.16 ^b^	0.61 ± 0.00 ^d^	1.16 ± 0.00 ^c^
31		Bicyclo[2.1.0]pentane, 1,4-dimethyl-	17065-18-8	-	3.96 ± 0.58 ^c^	17.62 ± 0.00 ^a^	14.49 ± 0.00 ^b^
32		Pentane, 1-chloro-	543-59-9	-	-	4.63 ± 5.22	15.99 ± 1.87
33	**ketones**	1-Penten-3-one	1629-58-9	93.46 ± 12.41 ^a^	19.40 ± 4.27 ^b^	3.93 ± 1.67 ^b^	2.30 ± 0.46 ^b^
34		3,5-Octadien-2-one	38284-27-4	38.27 ± 4.85 ^a^	10.09 ± 0.00 ^b^	10.04 ± 0.00 ^b^	5.93 ± 0.22 ^b^
35		3-Octen-2-one, (E)-	18402-82-9	-	2.86 ± 0.00 ^b^	1.45 ± 0.00 ^b^	23.40 ± 2.31 ^a^
36		2-Methylhept-6-en-3-one	26144-88-7	-	-	22.85 ± 0.00	-
37	**aldehydes**	4-Ethyl-2-hexynal	71932-97-3	72.81 ± 0.00 ^a^	-	5.45 ± 0.00 ^b^	-
38		Hexanal	66-25-1	6504.75 ± 25.11 ^a^	2936.55 ± 31.74 ^b^	730.45 ± 39.90 ^c^	613.60 ± 7.12 ^c^
39		Nonanal	124-19-6	54.53 ± 9.10 ^a^	30.02 ± 3.06 ^b^	36.51 ± 34.53 ^b^	69.90 ± 4.95 ^a^
40		2,4-Hexadienal, (E,E)-	142-83-6	51.48 ± 0.12 ^a^	12.46 ± 2.40 ^b^	5.51 ± 0.00 ^c^	
41		Pentanal	110-62-3	45.89 ± 3.08 ^a^	18.87 ± 0.55 ^b^	14.84 ± 12.50 ^b^	30.86 ± 2.78 ^ab^
42		Heptanal	111-71-7	45.17 ± 48.18	39.97 ± 6.05	35.71 ± 5.40	38.59 ± 0.37
43		2-Pentenal, (E)-	1576-87-0	40.08 ± 5.37	-	-	-
44		2-Heptenal, (E)-	18829-55-5	31.82 ± 0.60 ^a^	18.18 ± 1.47 ^b^	10.91 ± 8.97 ^b^	44.21 ± 1.28 ^a^
45		2,2-Dimethyl-4-octenal	30390-60-4	29.05 ± 0.00	-	-	-
46		2-Hexenal	505-57-7	2807.95 ± 57.59 ^a^	2138.64 ± 35.97 ^a^	844.93 ± 43.86 ^b^	881.93 ± 8.41 ^b^
47		1-Cyclohexene-1-carboxaldehyde, 2,6,6-trimethyl-	432-25-7	25.72 ± 1.54 ^a^	6.51 ± 0.18 ^b^	2.37 ± 0.00 ^c^	1.86 ± 0.10 ^c^
48		2-Octenal, (E)-	2548-87-0	203.26 ± 14.91 ^a^	56.55 ± 51.25 ^b^	21.96 ± 8.12 ^b^	17.34 ± 0.84 ^b^
49		2-Hexenal, (E)-	6728-26-3	16.95 ± 0.00 ^b^	38.97 ± 0.00 ^a^	11.15 ± 5.09 ^b^	10.95 ± 0.00 ^b^
50		2,6-Nonadienal, (E,Z)-	557-48-2	140.95 ± 14.00 ^a^	17.53 ± 0.22 ^b^	8.27 ± 1.63 ^b^	4.07 ± 0.01 ^b^
51		2,4-Heptadienal, (E,E)-	4313-03-5	139.46 ± 16.53 ^a^	27.96 ± 1.30 ^b^	4.01 ± 0.50 ^b^	-
52		2-Nonenal, (E)-	18829-56-6	100.98 ± 0.16 ^a^	30.24 ± 2.17 ^b^	17.35 ± 3.30 ^c^	10.73 ± 0.63 ^d^
53	**acids**	Propanoic acid, anhydride	123-62-6	27.71 ± 10.85	-	-	-
54		Pentanoic acid, 2-methyl-, anhydride	63169-61-9	-	4.59 ± 0.00 ^a^	3.60 ± 0.00 ^b^	-
55		Acetic acid, hydroxy-	79-14-1	18.42 ± 0.00 ^a^	-	-	1.86 ± 0.00 ^b^
56	**Others**	1,3-Hexadiene, 3-ethyl-2-methyl-	61142-36-7	187.92 ± 10.01 ^a^	98.36 ± 2.56 ^b^	24.14 ± 17.84 ^c^	-
57		7-Oxabicyclo[4.1.0]heptane	286-20-4	-	32.45 ± 0.00 ^a^	-	11.73 ± 0.16 ^b^
58		3-Methoxyhex-1-ene	108811-41-2	-	20.68 ± 0.00	-	-
59		Cyanamide, dimethyl-	1467-79-4	-	364.72 ± 0.00	-	-
60		Furan, 2-pentyl-	3777-69-3	54.98 ± 7.84 ^a^	21.25 ± 2.06 ^b^	9.60 ± 10.43 ^b^	3.55 ± 0.23 ^b^
61		Furan, 2-butyltetrahydro-	1004-29-1	52.40 ± 1.21 ^a^	25.50 ± 0.00 ^b^	5.37 ± 0.00 ^c^	9.20 ± 7.97 ^c^
62		trans-2-(2-Pentenyl)furan	70424-14-5	23.25 ± 0.00	-	-	-
63		Furan, 2-ethyl-	3208-16-0	20.09 ± 0.77 ^a^	5.08 ± 0.12 ^b^	1.34 ± 0.54 ^c^	6.23 ± 0.26 ^b^
64		1,1′-Biphenyl, 4-methoxy-	613-37-6	12.76 ± 0.33 ^a^	4.32 ± 0.36 ^b^	0.95 ± 0.34 ^c^	1.04 ± 0.13 ^c^
65		Butane, 1-(2-propenyloxy)-	3739-64-8	202.81 ± 9.94 ^a^	20.46 ± 0.00 ^b^	3.62 ± 1.98 ^c^	4.69 ± 0.58 ^c^
66		1H-1,2,4-Triazole	288-88-0	12.43 ± 0.00	-	-	-
67		1-Methyl-1H-1,2,4-triazole	6086-21-1	-	-	15.67 ± 0.00 ^a^	0.84 ± 0.00 ^b^

Note: Data in the table are expressed as means ± standard deviation. For each line, different lowercase letters correspond to values significantly different at *p* < 0.05. - indicates not annotated.

**Table 6:** Sensory evaluation standard of golden mulberry.

Marking Scheme	Color and Luster	Juicy	Sweet	Sour	Flavor
0–4.9	Light green, indicating lack of luster	Largely absent	Astringent	Sour and astringent	No fruity flavor
5.0–9.9	Greenish, dull surface	Not much	Slightly sweet	Slightly acidic	Lightly fruity
10–14.9	Greenish yellow, brightly colored	More	Sweet	suitably sweet and sour	Strong flavor, milk flavor
15–20	Yellowish white, bright surface	Rich	Very sweet	No acidity	Stronger aroma, sweeter

## Data Availability

Data will be provided upon request.

## Abbreviations

ABTS	2,2′-azino-bis(3-ethylbenzothiazoline-6-sulfonic acid)
Chromaticity	C*
DPPH	1-diphenyl-2-picrylhydrazyl radical
FRAP	ferric reducing antioxidant power
GAE	gallic acid equivalent
SD	standard deviation
SSs	soluble sugars
TSSs	total soluble solids
TA	titratable acidity
